# Microstructure of magnesium silicate hydrate pastes influenced by carbonate and mixing method

**DOI:** 10.1617/s11527-025-02837-0

**Published:** 2025-10-23

**Authors:** M. H. N. Yio, E. Bernard, C. Dewitte, H. Chen, T. C. Chan, R. J. Myers

**Affiliations:** 1 Department of Civil and Environmental Engineering, Imperial College London, London, SW7 2AZ UK; 2 Empa, Swiss Federal Laboratories for Materials Science and Technology, Laboratory for Concrete and Asphalt, 8600 Dübendorf, Switzerland

**Keywords:** Magnesium silicate hydrate (M-S-H), Microstructure, Porosity, Brucite, Mechanical performance

## Abstract

This study investigates the microstructure of magnesium silicate hydrate (M-S-H) pastes prepared with MgO and silica fume from different sources but of comparable properties, with varying sodium carbonate (NC) contents, using two mixing methods: conventional Hobart-style paddle mixing and low-speed ball-mill mixing. The latter method significantly improved silica fume dispersion and particle packing, resulting in a denser microstructure and substantially higher compressive strength compared to conventional mixing. A NC content of 2.5 wt.% was identified as optimal for enhancing microstructure and reducing porosity in conventionally mixed samples, while no clear effect of NC content observed in ball-milled ones. Brucite was detected in NC-containing samples by thermogravimetric analysis, but not by X-ray diffraction, indicating its poor crystallinity. Compressive strength increased with M-S-H formation and decreasing brucite content. These findings highlight the importance of optimising particle packing for improving the microstructure and mechanical properties of magnesium silicate binders.

**Supplementary Information:**

The online version contains supplementary material available at 10.1617/s11527-025-02837-0.

## Introduction

Magnesium-silicate hydrate (M-S-H), originally investigated as a byproduct of cement degradation in contact with magnesium-rich environments [1, 2], has gained increasing attention as a potential low-carbon binder for a wide range of civil engineering applications. These include specialised uses such as nuclear waste encapsulation [3, 4] and stabilisation of hazardous wastes [5], owing to its low pH and promising mechanical properties [6]. In contrast to Portland cement (PC), which relies on carbonate rocks (i.e. limestone) for CaO production, magnesium-silicate binders can be produced from non-carbonate rocks such as serpentine and olivine. Ongoing research is focused on developing scalable, low carbon technologies to extract reactive MgO and amorphous silica from magnesium-silicate rocks [7], and MgO from brines [8] and seawater [9].

These binders are typically produced by hydrating reactive MgO with silicate sources such as silica fume [10–12]. The process begins with MgO hydration to form Mg(OH)_2_, which then reacts with reactive SiO_2_ to form M-S-H as the primary binding phase [13]. The process is slow but can be accelerated with the use of additives, such as phosphates and carbonates [14–17], although their effects on the microstructure remain poorly understood. Depending on the Mg/Si ratio and water-to-binder (w/b) ratio, unreacted MgO, residual silica, and Mg(OH)_2_ can remain in the system [14, 18].

Extensive research has been carried out on the hydration chemistry and properties of M-S-H [10–12, 19, 20]. Similar to calcium-silicate-hydrate (C-S-H) in PC systems, M-S-H exhibits variable composition. For instance, M-S-H phases formed in degraded PC systems tend to have a low Mg/Si ratio (< 0.8). Recent studies have reported M-S-H with an Mg/Si ratio as low as 0.67 (comparable to that of sepiolite, a Mg-Si phyllosilicate) following carbonation [21], and as high as 1.5 (similar to that found in serpentinite group) in the presence of Na_2_CO_3_ [16]. Compared to natural Mg-Si phyllosilicate minerals, M-S-H contains more water and exhibits a more nano-crystalline structure [10, 20].

In terms of performance, magnesium-silicate binders generally exhibit good mechanical strength, but reported values often show large variability despite similar compositions and reaction extents. While there is a general trend with w/b ratio [22], other important factors, such as physical characteristics of raw materials and their effects on the microstructure have been less studied. For instance, Tran et al. [23] reported improved performance at a high Mg/Si ratio of 2.25 and highlighted the role of particle packing density, influenced by the size and shape of the raw materials, but did not quantitatively investigate the microstructure. Durability aspects of M-S-H binders also remain under-investigated. Shah et al. [24] found that using metakaolin over silica fume resulted in superior transport properties, but the underlying link between raw material properties and M-S-H binder microstructure remains unclear.

When silica fume (the most studied silicate source) is used, the presence of large agglomerates or clusters [25] can significantly impact the homogeneity and density of the microstructure and in turn the overall performance. Similar issues have been observed in PC systems blended with silica fume [26], where pre-dispersion in slurry form or by high-speed mixing and ultrasonication have proven beneficial [27]. While such agglomerates may be broken down in concrete systems due to the shearing action induced by aggregate particles during mixing, they pose challenges in paste and mortar systems which remain important for fundamental research.

As a follow-up of Bernard et al. [17], which investigated the effects of carbonate and phosphate on M-S-H hydration, this study focuses on the microstructural effects. It employs two mixing techniques to prepare M-S-H pastes using MgO and silica fume from different sources but with comparable properties, at varying Na_2_CO_3_ concentrations: (1) conventional Hobart-style paddle mixing without pre-dispersion of silica fume, and (2) low-speed ball-mill mixing aimed at improving silica fume dispersion. Systematic characterisation of the microstructure, phase assemblage, and compressive strength of these M-S-H pastes are reported here, including analysis of the correlations between these properties.

## Experimental

### Materials

The raw materials for conventional and ball-mill mixing were sourced from different suppliers, due to adjustments to the original research plan. For conventional mixing, MgO (CalMag 92/200) from RBH UK and densified silica fume (Microsilica 920) from Elkem were used. For ball-mill mixing, a 50/50 wt.% MgO blend was used, consisting of MgO produced by calcining Mg(OH)_2_ (Sigma-Aldrich) at 600 °C for 2 h (with the temperature increased gradually from room temperature to 600 °C to avoid spattering) and commercial MgO (Baymag), along with undensified silica fume (Microsilica 968, Elkem) to further promote dispersion. Sodium carbonate (NC) (anhydrous, ≥ 98% purity, VWR) and sodium hexametaphosphate (NHMP) (general purpose grade, Fisher Scientific) were used as an accelerator and a superplasticiser, respectively. Despite the different sources of raw materials, they shared comparable compositions, physical properties and reactivity, as shown in Table 1 and Fig. S1 (Supplementary Information). Therefore, their influence is expected to be minimal compared with the effect of mixing method, and comparisons between the two methods remain meaningful.

**Table 1:** Chemical compositions and physical properties of raw materials

	MgO		Silica fume		NHMP
	Conventional mixing	Ball-mill mixing	Conventional mixing	Ball-mill mixing	
*XRF oxide composition (wt.%)*					
SiO_2_	2.05	0.12	94.23	96.1	< 0.11
Al_2_O_3_	0.38	< 0.11	0.22	0.23	< 0.11
Fe_2_O_3_	0.86	0.34	0.81	0.06	< 0.04
Cr_2_O_3_	< 0.003	< 0.003	< 0.003	< 0.003	< 0.003
MnO	0.070	< 0.013	0.082	0.028	< 0.004
TiO_2_	< 0.019	< 0.019	< 0.019	< 0.019	< 0.019
P_2_O_5_	0.148	< 0.017	0.033	< 0.017	66.85
CaO	1.95	0.74	0.18	0.30	< 0.05
MgO	86.2	97.1	0.85	0.38	< 0.08
K_2_O	0.05	< 0.03	0.43	0.79	0.09
Na_2_O	< 0.06	0.10	0.42	0.17	31.9
SO_3_	0.20	0.42	0.06	0.19	0.07
L.O.I	8.05	1.09	2.65	1.57	1.00
Total	99.96	99.90	99.95	99.94	99.93
TC	0.62	n.a	1.33	n.a	0.05
*Physical properties*					
Laser granulometry d_50_ (µm)	12.9 ± 0.2	13.4 ± 0.4	34.6 ± 1.2	23.8 ± 0.2	n.a
BET specific surface area (m^2^/g)	21.5 ± 0.2	24.0	22.7 ± 0.3	20.9	n.a
He pycnometry specific gravity (-)	3.0	3.5	2.2–2.3	2.3	n.a
Citric acid reactivity (s)	88	105	n.a	n.a	n.a

### Sample preparation

The mix designs are presented in Table 2. The notation MS_*x* refers to samples prepared by conventional mixing, where *x* indicates the NC concentration. Similarly, MSb_*x* refers to samples prepared by ball-mill mixing, with ‘b’ denoting the ball-mill method. All mixes were prepared with a constant Mg/Si molar ratio of 1.5 and a w/b mass ratio of 0.7. NC was added at 0, 1, 2.5 and 5 wt.% of the binder, while NHMP was incorporated at varying concentrations from 0.2 to 2 wt.% to ensure comparable workability across mixes.

**Table 2:** Mix designs. SF = silica fume, NC = sodium carbonate, NHMP = sodium hexametaphosphate

Sample ID	MgO (wt.%)	SF (wt.%)	NC		Water (wt.%)	NHMP		w/b	Mg/Si
			(wt.%)	(wt.% binder)		(wt.%)	(wt.% binder)		
MS_0	29.4	29.4	–	–	41.1	0.1	0.2	0.70	1.5
MS_1	29.1	29.1	0.6	1.0	40.7	0.6	1.0	0.69	1.5
MS_2.5	28.6	28.6	1.4	2.5	40.1	1.2	2.0	0.68	1.5
MS_5	28.2	28.2	2.8	5.0	39.6	1.1	2.0	0.67	1.5
MSb_0	29.4	29.4	-	-	40.6	0.6	1.0	0.69	1.5
MSb_2.5	28.7	28.7	1.4	2.5	40.5	0.6	1.0	0.71	1.5

For conventional mixing (hereafter referred to as ‘mixing’ or ‘mixed’ for brevity), MgO and silica fume (SF) were homogenised using a high-speed powder blender for 2 min. NHMP and NC were pre-dissolved in deionised water. The liquid solution was then added to the homogenised powder in a 30 L Hobart mixer and mixed using a ‘B’-style paddle attachment for 3 min. The mixes were vibrated and compacted in three layers in stainless steel moulds (50 × 50 × 50 mm^3^) and covered with a plastic sheet. After 24 h, the samples were demoulded and cured at 21 °C, 98% RH for 3, 7, 28, 91 days. For each mix and curing age, four cubes were prepared, three for compressive strength (procedure described in [17]) and one for microanalyses, from which two prisms of 40 × 20 × 8 mm^3^ were extracted, one for backscattered electron (BSE) microscopy and the other for water absorption, with the remainder crushed into granules and powder for other analyses.

For ball-mill mixing (hereafter referred to as ‘ball-milling’ or ‘ball-milled’), MgO and SF were first homogenised in a Turbula shaker mixer for 1 h. NHMP and NC were pre-dissolved in deionised water, and the resulting solution was added to the homogenised powder in a 250 ml tungsten carbide jar containing 10 balls of 12 mm, and mixed at 250 rpm for 5 min. The mixing speed and duration were intentionally kept low to promote SF dispersion without causing comminution of the particle sizes. The wet mixture was then cast into steel moulds (25 × 25 × 25 mm^3^) to form three cubes for compressive strength, and into a plastic container (37 mm diameter) to produce a cylinder for BSE imaging and other microanalyses. The samples were demoulded and cured similarly to the mixed samples, but only one curing age (i.e. 91 days) was considered.

The samples for BSE and microanalyses were hydration-stopped by immersing them in isopropanol at a volumetric ratio of 1:15, followed by diethyl ether for 5 min. The samples were then oven dried at 40 °C for a few hours, details can be found in [17]. This method is commonly used for PC systems, but it is acknowledged that its feasibility and potential to introduce artefacts in magnesium silicate cements have not yet been systematically studied and require further investigation. However, it is worth noting that based on prior experience with freeze-drying M-S-H suspensions containing brucite [16], this method does not appear to modify the chemical composition of either brucite or M-S-H.

The extracted samples for BSE imaging were epoxy-impregnated and surface ground to 0.25 µm fineness and carbon-coated. The remaining samples were crushed into granules of a few mm for MIP and N_2_ sorption analyses, or ground into powder for TGA, XRD and ^29^Si NMR analyses. Detailed descriptions and data for XRD and ^29^Si NMR analyses of the mixed samples are provided in [17]; the same measurements were repeated for the ball-milled samples in this study.

### Characterisation

#### Thermogravimetry analysis (TGA)

TGA was performed using a Netzsch STA 449 F5 Jupiter analyser, with ~ 60 mg of powdered sample placed in an alumina crucible and heated from 30 °C to 950 °C at a rate of 20 °C/min under a N_2_ flow of 50 ml/min. The total bound water was determined as the mass difference between 40 °C and 950 °C. Chemically bound water in brucite (CBW_brucite_) was calculated from the derivative curve using the tangential method [28] within 360–470 °C, and the corresponding brucite content was derived using the molecular masses of brucite and water. Chemically bound water (CBW_M-S-H_) in M-S-H was quantified as the mass loss between 250 °C and 950 °C, after subtracting CBW_brucite_. It should be noted this temperature range may also include the decomposition of carbonates, potentially causing a slight overestimation of CBW_M-S-H_. However, phase assemblage results (Sect. 3.1) indicate that carbonate from NC was most likely incorporated into brucite and thus already accounted for as part of CBW_brucite_.

Physically adsorbed water (PAW_M-S-H_) was then calculated by subtracting CBW_M-S-H_ and CBW_brucite_ from the total bound water. The total water in M-S-H was taken as the sum of PAW_M-S-H_ and CBW_M-S-H_. All results were normalised to 100 g anhydrous material by scaling to the mass remaining at 950 °C for fair comparisons. Two values were corrected by applying a factor of 3.25 (see Fig. S2) to the measured CBW. The original values were identified as outliers, due to the measured PAW_M-S-H_ being inconsistent, likely due to its sensitivity to drying and storage conditions [11].

#### Fourier transform infrared spectroscopy (FTIR)

FTIR analysis was conducted using a Thermo Scientific Nicolet iS50 FTIR spectrometer in the attenuated total reflectance (ATR) mode. Spectra were collected over the 400–4000 cm^−1^ range with a spectral resolution of 4 cm^−1^, averaging 32 scans per sample.

#### Mercury intrusion porosimetry (MIP)

MIP was conducted using a Quantachrome Poremaster 60 on 7-day and 91-day samples. Approximately 1 g of granules was de-aired in a penetrometer and subsequently filled with mercury under increasing pressure, first up to 0.34 MPa and then to 414 MPa. This pressure range covered pore entry diameters from 4 nm to > 100 µm based on the Washburn equation, assuming a mercury contact angle of 140° and a surface tension of 0.8 N/m. Three main pore parameters were analysed: porosity represented as the total intruded pore volume, the critical pore size identified from the largest peak in the differential pore size distribution curve, and the median pore size from the cumulative distribution curve.

#### Apparent water absorption

For the mixed samples, specimens were submerged in water under vacuum for ~ 2 h to achieve full saturation, and the apparent water-accessible porosity was determined based on the mass increase. Sorptivity measurement was further conducted on the 91-day samples by placing them vertically in a tray with a 2 mm depth of water to determine their rate of absorption. The mass of water absorbed was measured at regular intervals using an electronic balance of 0.001 g accuracy. The results were plotted as cumulative absorption per unit inflow area against the square root of time, and the slope of regression line (R^2^ ≥ 0.99) was taken as the sorptivity coefficient. In contrast, the ball-milled samples cracked and disintegrated immediately upon contact with water (see Sect. 3.2.1). Therefore, water absorption measurements could not be performed.

#### ***N***_***2***_*** adsorption***

N_2_ adsorption analysis was performed using a Micromeritics 3Flex 3500 analyser. Approximately 1 g of crushed granules of a few mm in size were degassed externally on a VacPrep 061 degassing unit at 40 °C under vacuum for 24 h prior to measurement. This protocol was chosen based on established procedures for PC-based systems [29]. While additional vacuum drying could potentially alter the microstructure, it is necessary to ensure that the pores are properly degassed.

Brunauer–Emmett–Teller (BET) analysis [30] was performed in the relative pressure (p/p₀) range selected based on the maximum of the Rouquerol [31], mostly within 0.05–0.25, to determine the pore specific surface area (SSA). The total pore volume (V) was determined based on N_2_ adsorption at a p/p₀ of 0.99 and was further classified into three sizes: < 2 nm, 2–50 nm, and > 50 nm. Pore volume < 2 nm was estimated by means of t-plot analysis, using the layer thickness of non-porous silica nanoparticles and microporous silica [32]. Pore volume in the 2–50 nm range was analysed according to the Barrett-Joyner-Halenda method [33], while the remaining pore volume was assigned to > 50 nm. Furthermore, the mean pore diameter was calculated as 4 V/SSA [34], assuming a cylindrical shape.

#### Backscattered electron (BSE) microscopy

BSE imaging was performed on 91-day samples containing 0 and 2.5 wt.% NC using a Zeiss Sigma 500 VP SEM at an accelerating voltage of 15 kV and a working distance of 8.5 mm. The magnification was set to 500 × to achieve a pixel spacing of 0.223 µm. Porosity was estimated by histogram-based manual thresholding. Energy dispersive spectroscopy (EDS) mapping was conducted on the 2.5 wt.% NC samples using an Oxford Instruments Aztec Energy Advanced Xmax 150 detector to observe the spatial distribution of Mg, Si and other elements, with calibration performed by measuring pure cobalt. The acquired maps (with a resolution of 512 × 384 pixels and a dwell time of 10 to 15 ms, averaged over 20–30 frames) were analysed using Aztec QuantMap. Oxygen was quantified by stoichiometry, and the results were expressed as atomic Mg/Si ratios.

## Results and discussion

### Phase assemblage

Table 3 presents the amounts of brucite, total water in M-S-H, and total bound water, as quantified by TGA (see Fig. S3 for 3-day and 91-day curves). For the mixed samples, a clear increase in total water in M-S-H was observed with both age and NC, with the effect of NC particularly notable between 2.5 wt.% and 5 wt.%. On average, more than half of the water in M-S-H (~ 65%) was physically bound (Table S1), and this proportion appears to increase with NC. A clear reduction in brucite was observed with both increasing age and incorporation of NC (even at 1 wt.%). For ball-mill mixing samples, the effect of NC at 2.5 wt.% was particularly evident, resulting in higher total water in M-S-H.

**Table 3:** Quantification of brucite, total water in M-S-H and total bound water from TGA data. All results were normalised to 100 g anhydrous materials. The typical relative error is between ± 5% and ± 10% [28]. MS_x: conventional mixing; MSb_x: ball-mill mixing (x = NC concentration)

Sample ID	Brucite^a,b^				Total water in M-S-H^a^				Total bound water			
	3d	7d	28d	91d	3d	7d	28d	91d	3d	7d	28d	91d
MS_0	21.6	16.7	14.6	8.93	16.7	22.4	33.9	42.4	23.4	27.6	38.4	45.2
MS_1	11.0	12.0	8.23	5.41	24.2	26.9	35.6	42.3	27.6	30.6	38.1	44.0
MS_2.5	13.6	10.9	8.40	5.86	26.3^c^	37.3	43.1	46.6	30.2^d^	40.7	45.7	48.4
MS_5	10.4	10.4	7.23	5.47	30.8	28.0	37.4	50.1^c^	34.0	31.2	39.7	51.9^d^
MSb_0	–	–	–	10.5	–	–	–	32.6	–	–	–	35.8
MSb_2.5	–	–	–	9.31	–	–	–	43.9	–	–	–	46.8

^a^CBW_brucite_, PAW_M-S-H_ and CBW_M-S-H_ are provided in Table S1

^b^Accounts for both crystalline and poorly crystalline brucite

^c^Estimated from Fig. S4 as 3.25 × CBW_M-S-H_

^d^Recalculated as the sum of estimated total water in M-S-H and CBW_brucite_

For the carbonate-free samples (both mixed and ball-milled), the amount of brucite quantified by TGA agreed well with the content determined by XRD Rietveld analysis (Fig. S3, Fig. S4a [17]), suggesting the presence of a single crystalline form of brucite. However, in the samples containing carbonates, the brucite content measured by TGA was higher than that detected by XRD, where crystalline brucite was barely observable. This observation is more clearly illustrated in Fig. S5 which plots the brucite content obtained from XRD against that from TGA. This discrepancy is attributed to reduced brucite crystallinity, i.e. the formation of nano- or poorly crystalline brucite, potentially related to the incorporation of carbonates and water into its main layers, forming hydrous carbonate-containing brucite (HCB) [35, 36].

**Fig. 1:**
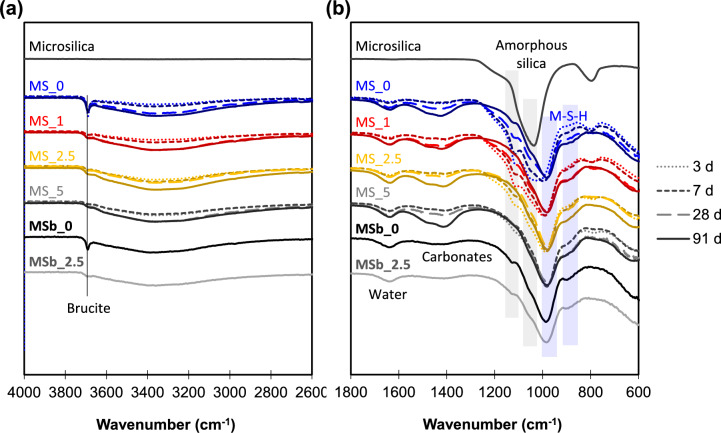
FTIR spectra shown in two magnified regions **a** 2600–4000 cm^−1^ and **b** 600–1800 cm^−1^. MS_x: conventional mixing; MSb_x: ball-mill mixing (x = NC concentration)

Figure 1 presents the FTIR data for the mixed samples. In line with ^29^Si NMR measurements (Fig. S4b, Table S2, [17]), a noticeable decrease in unreacted SF was observed over time and with the incorporation of NC, as indicated by the diminishing peak at 1050 cm^−1^ associated with amorphous silica. At the same age, the amount of water and carbonates appeared similar across all samples, regardless of NC content. Consistent with the XRD data, only a trace amount of crystalline brucite was detected in the carbonate-containing samples, indicated by the weak band at 3698 cm^−1^ [37]. This suggests that the brucite observed by TGA was most likely poorly crystalline brucite, as discussed above.

**Fig. 2:**
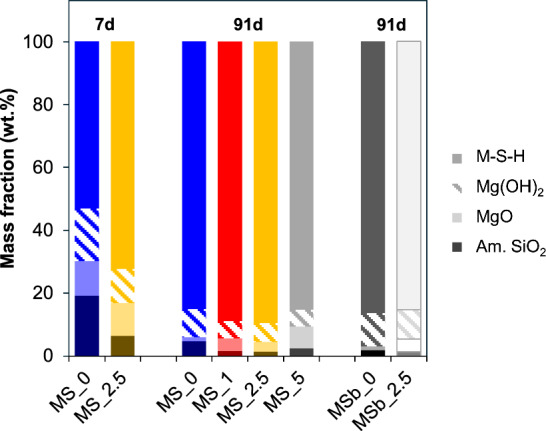
Phase assemblages estimated based on results from.^29^Si MAS NMR (for unreacted silica fume, Am. SiO_2_), TGA (for Mg(OH)_2_, both crystalline and poorly crystalline brucite), and XRD Rietveld analysis (for unreacted MgO). M-S-H was estimated from mass balance. Considering uncertainties in measurements and calculations, the overall error is estimated at ± 10%. MS_x: conventional mixing; MSb_x: ball-mill mixing (x = NC concentration**)**

Based on the results from ^29^Si MAS NMR, XRD and TGA, the overall solid phase assemblage at 7 and 91 days were calculated, as shown in Fig. 2. At 7 days, as NC increased from 0 to 2.5 wt.%, unreacted MgO in the mixed samples decreased from 19.2 wt.% to 6.5 wt.%, and brucite (crystalline and poorly crystalline, from TGA) declined from 16.7 wt.% to 10.9 wt.%, while M-S-H increased from 50 wt.% to 70 wt.%. By 91 days, the mixed samples exhibited phase compositions comparable to those of the ball-milled samples. This is attributed to the relatively high w/b ratio used, allowing for maximised reactions in both mixed and ball-milled samples. MS_0 and MSb_0 contained 4.7 wt.% and 1.9 wt.% MgO, and 8.9 and 10.5 wt.% crystalline brucite, respectively. In contrast, MS_2.5 and MSb_2.5 both contained 1.4 wt.% MgO, and 5.9 wt.% and 9.3 wt.% brucite (crystalline and poorly crystalline, from TGA), respectively. M-S-H accounted for more than > 85 wt.% in all samples.

### Microstructure

#### MIP and water-accessible porosity

**Fig. 3:**
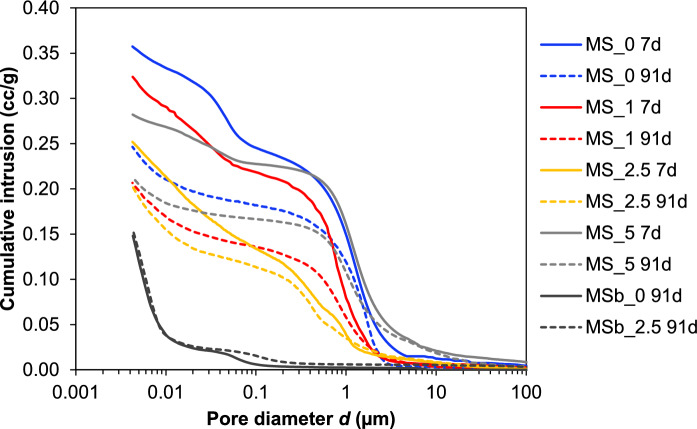
Pore size distribution measured by MIP. MS_x: conventional mixing; MSb_x: ball-mill mixing (x = NC concentration)

Figure 3 shows the pore size distributions measured using MIP, with the cumulative intrusion volume, critical and median pore diameters presented in Table 4. Both porosity and d_median_ decreased with increasing NC, reaching a minimum at 2.5 wt.% NC. Beyond this point, both parameters increased again at 5 wt.% NC, suggesting that 2.5 wt.% NC promotes maximum formation of hydrates to fill the pore space. This is consistent with the hydration chemistry results presented in [17], which show that excessive sodium carbonate reduces the degree of reaction of MgO and silica. The effect of carbonate was particularly significant at an early age of 7 days, where porosity and d_median_ at 2.5 wt.% NC were 30% and 80% lower compared to 0 wt.% NC, respectively. However, this effect became less pronounced at 90 days.

**Table 4:** Cumulative mercury intrusion, critical (*d*_crit_) and median (*d*_median_) pore diameters measured by MIP. MS_x: conventional mixing; MSb_x: ball-mill mixing (x = NC concentration)

Sample ID	Cumulative intrusion (cc/g)		*d*_crit_ (µm)		*d*_median_ (µm)	
	7d	91d	7d	91d	7d	91d
MS_0	0.357	0.246	1.242	1.736	0.718	0.922
MS_1	0.324	0.206	0.769	0.805	0.549	0.459
MS_2.5	0.252	0.203	1.055	0.447	0.141	0.206
MS_5	0.282	0.212	1.167	1.048	1.110	0.982
MSb_0	–	0.148	–	0.059	–	0.007
MSb_2.5	–	0.154	–	0.109	–	0.007

In contrast, the ball-milled samples exhibited much lower porosity and smaller pore sizes compared to the mixed samples. However, there was no clear effect of increasing carbonate content. At 0 wt.% NC, the porosity in the ball-milled sample was 40% lower than that of the mixed sample, while at 2.5 wt.% NC, the porosity was 24% lower. The d_median_ was 100% lower in the ball-milled samples than in the corresponding mixed samples at both NC contents. Although the raw materials for the conventionally mixed and ball-milled samples were different, their properties were comparable, with the MgO showing similar reactivity, and the silica fume having comparable purity and particle size distributions (Table 1, Fig. S1). While contributions from raw material differences cannot be entirely ruled out, the observed effects are attributed mainly to the mixing method.

Table 5 presents the apparent water absorption results, including the water-accessible porosity and sorptivity for the mixed samples. In general, the results align with those from MIP, showing that porosity reduced with increasing NC content up to 2.5 wt.%. This effect was particularly significant at 7 days, where porosity decreased by 44% between 0 and 2.5 wt.% NC. The porosity also reduced consistently with age, indicating that the formation of M-S-H progressively filled the pore space. However, the results appear to contradict the findings of Shah et al. [24], which reported that porosity remained constant over time. This discrepancy may be attributed to the differences in the drying methods used: solvent exchange (milder) in this study versus oven drying (harsher and can potentially induce cracking [38]) in [24]. This is supported by the lower porosity values observed in this study, despite the use of higher w/b ratios. In line with the porosity results, the sorptivity at 91 days was lowest for the 2.5 wt.% NC sample. The cumulative absorption curves are available in Fig. S6.

**Table 5:** Apparent water porosity after vacuum saturation, sorptivity and coefficient of determination (R^2^). The typical relative error is ± 5% [38]. MS_x: conventional mixing (x = NC concentration)

Sample ID	Apparent water porosity (cc/g)				Sorptivity (cc/g∙min^0.5^)		R^2^
	3d	7d	28d	91d	91d		
MS_0	43.5	41.0	33.5	24.7	1.270	0.995	
MS_1	36.1	33.7	25.2	17.7	0.938	0.994	
MS_2.5	35.6	23.1	24.0	18.1	0.894	0.987	
MS_5	29.1	26.8	24.4	21.7	1.027	0.989	

Fig. S7 compares the measured MIP and apparent water-accessible porosity for 91-day samples with thermodynamically modelled results [17]. For the mixed samples, the measured porosities were generally lower than the modelled values but followed a similar trend, with lower porosities observed between 1 and 2.5 wt.% NC. These results suggest that some free water may still be present in the pore structure of the solvent exchanged-samples. However, given that all samples were dried in the same way, the results are deemed relative and comparable across the different NC concentrations. The ball-milled samples exhibited significantly lower porosity as measured by MIP; however, the effect of NC content was not apparent in these samples. This can be attributed to the already very dense microstructure induced by ball milling, which enhances particle packing and dispersion. Such densification likely restricts the mobility of Mg ions, thereby limiting further reaction progression (Sect. 3.2.3), particularly under the equilibrium pH conditions of ~ 10 [16], even in the presence of NC.

The disintegration of the ball-milled samples upon contact with water is a significant phenomenon and warrants further investigations. This could be linked to significant shrinkage [39] and expansion of M-S-H upon drying and wetting. The relatively dense microstructure provided no space for stress release, unlike the mixed samples, which were inherently more porous. A similar phenomenon was observed in a separate paste sample prepared with a different MgO and well-dispersed SF (Fig. S8), indicating that this behaviour is reproducible and may limit the practical use of such binders, particularly in environments where contact with water is expected. This issue could potentially be mitigated using shrinkage-reducing admixture (SRA), as described in [40], although this may come at the expense of reduced strength. Alternatively, employing alternative silicate sources such as metakaolin, incorporating additional MgO to form excess brucite [41], or adding aggregates can provide restraint to counteract dimensional instability. Further work is necessary to understand better the underlying mechanisms and to improve the properties of such binders.

#### ***Pore specific surface area and volume from N***_***2***_*** sorption***

Table 6 presents the BET SSA and mean pore diameter measured using N_2_ adsorption. For the mixed samples, the addition of NC appears to result in larger SSA and smaller mean pore diameters, but no definitive trend was observed with increasing NC. With curing age, the BET SSA generally increased up to 28 days. This behaviour may be intrinsic, potentially indicating densification of the microstructure (e.g. agglomeration of M-S-H, as discussed in [11] on synthesised M-S-H). Alternatively, it could be related to insufficient drying or degassing, as free water becomes increasingly difficult to remove in more mature samples (also observed in PC systems [42]). However, the mean pore diameter consistently decreased with age, indicating densification of the microstructure. In contrast, the ball-milled samples showed lower BET SSA, but larger mean pore diameter compared to their mixed counterparts, possibly due to microcracking (see Sect. 3.2.3).

**Table 6:** BET specific surface area and mean pore diameter measured by N_2_ adsorption. The typical relative error is ± 10% [29]. MS_x: conventional mixing; MSb_x: ball-mill mixing (x = NC concentration)

Sample ID	BET SSA (m^2^/g)				C-value				BET mean pore diameter (nm)			
	3d	7d	28d	91d	3d	7d	28d	91d	3d	7d	28d	**91d**
MS_0	72	84	145	100	116	183	176	285	8.71	7.73	4.31	3.90
MS_1	110	149	129	120	157	154	148	178	5.90	5.18	4.57	3.23
MS_2.5	62	131	171	117	95	137	132	143	13.5	6.44	4.42	3.74
MS_5	87	149	149	116	100	156	141	176	7.22	4.12	3.48	2.76
MSb_0	–	–	–	88	–	–	–	120	–	–	–	5.86
MSb_2.5	–	–	–	53	–	–	–	126	–	–	–	7.25

Overall, the BET SSA values appear to align reasonably well with those reported for synthetic M-S-H aged for 3 years at 20 °C [11] (see Fig. S9). However, they are lower than those reported for paste samples prepared from colloidal silica and cured at 50 °C for > 50 days [25]. In general, the BET SSA values observed are higher than those reported for hydrated PC-based systems, which are typically < 100 m^2^/g [29]. This difference is attributable to the intrinsically higher SSA of M-S-H (~ 200 m^2^/g) compared to C-S-H (~ 120 m^2^/g) [43], and the presence of other phases in PC systems, including portlandite, AFm and AFt (with SSA of 8–10 m^2^/g [44]).

**Fig. 4:**
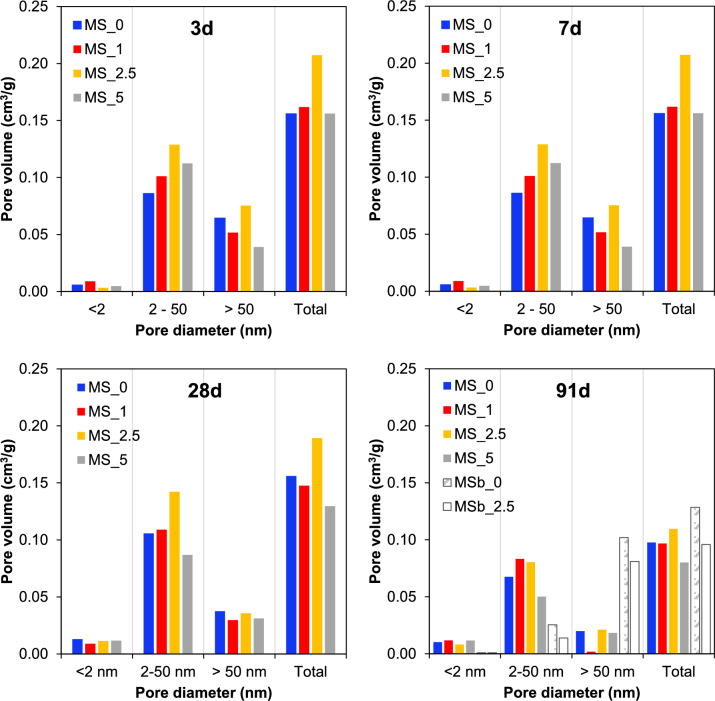
Classification of pores measured by N_2_ adsorption. The typical relative error is ± 10% [29]. MS_x: conventional mixing; MSb_x: ball-mill mixing (x = NC concentration)

Figure 4 shows the classification of pores measured by N_2_ adsorption. For the mixed samples, most pores fell within the 2–50 nm range, irrespective of NC content or curing age. The highest pore volumes were observed at 2.5 wt.% NC, both in the 2–50 nm and > 50 nm ranges, as well as the total pore volume. With increasing age, the total pore volume, and those in the 2–50 nm and > 50 nm ranges appeared to decrease, while those < 2 nm slightly increased, suggesting a refinement and densification of the pore structure. A similar observation was made by Martini et al. [45], who identified the presence of pores between 1–3 nm and 3–12 nm in MgO-SiO_2_ cement pastes (w/b = 2) cured for 28 days, using ^1^H NMR and low temperature differential scanning calorimetry. Interestingly, compared to the mixed samples, the ball-milled samples exhibited almost no pores < 2 nm, significantly lower volumes of pores in the 2–50 nm range, but higher volumes of pores > 50 nm. These observations are consistent with the mean pore diameters determined here (Table 6). The underlying reasons for these remain unclear, but they could be due to the formation of microcracks.

It should be noted that, since only a single measurement was performed, the observed differences between samples may fall within measurement uncertainty inherent to gas adsorption techniques [29]. Future research should include multiple replicate measurements to better assess variability and improve confidence in the results. In addition, further work is needed to optimise the degassing and testing protocols for analysing M-S-H pastes using N_2_ adsorption. Complementary techniques such as dynamic vapour sorption, which can probe finer pore structures, and ^1^H NMR, which does not require sample drying, can be further explored to provide a clearer picture of the pore structure at a finer scale.

#### BSE observation

**Fig. 5:**
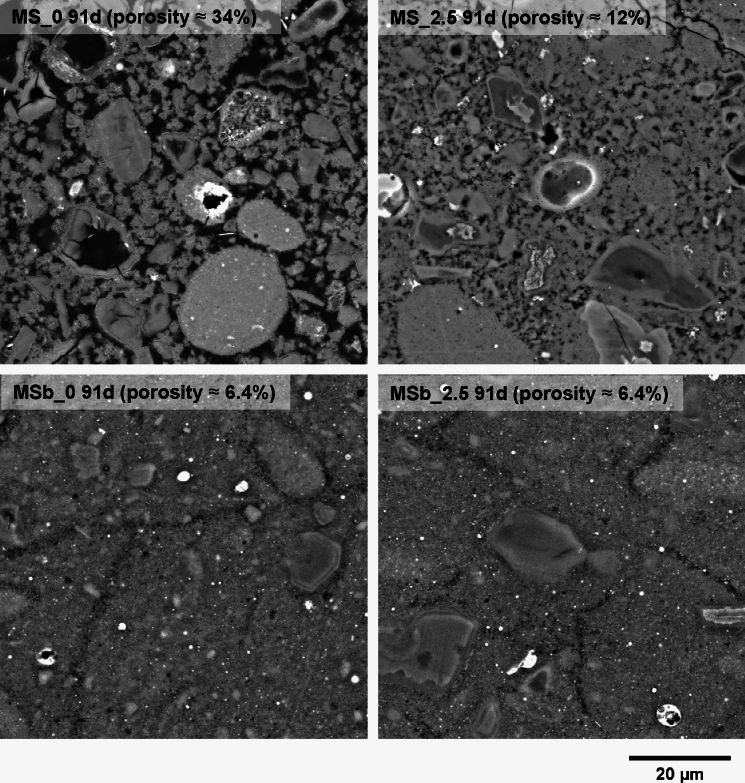
Representative BSE images showing the effects of sodium carbonate and preparation method on the microstructure of M-S-H pastes cured for 91 days. Porosity values (vol.%) obtained by histogram-based manual thresholding are indicated to aid comparison. MS_x: conventional mixing; MSb_x: ball-mill mixing (x = NC concentration)

Figure 5 shows representative BSE images of the mixed and ball-milled samples, with and without 2.5 wt.% NC at 91 days. Estimated porosity values are indicated for reference. In the mixed samples, a clear densification of the pore structure was observed with NC. At 0 wt.% NC, the sample appeared significantly porous, featuring large and interconnected pores in the order of a few microns. However, at 2.5 wt.% NC, the pores became more isolated, and hydrates with irregular morphology, likely M-S-H, clearly filled the originally water-filled spaces, forming a continuous matrix. MgO particles showed clear signs of reaction, as indicated by the darker grey level of the inner core, surrounded by a lighter shell, exhibiting the ‘Hadley’ grain effect (hollow hydration shells, some of which were filled with epoxy resin during sample preparation) commonly observed in PC-based systems [46]. There was also a clear presence of SF agglomerates, which was expected due to the mixing method used, resulting in poor dispersion.

Overall, the ball-milled samples appeared significantly denser, with a reduced presence of SF agglomerates, indicating effective dispersion. Most pores were in the submicron range, although microcracks of a few microns wide were observed across the samples without any specific pattern. These microcracks exhibited diffuse boundaries, suggesting they may be intrinsic, i.e. not induced by sample preparation. There was no clear effect of carbonate content on the pore structure, and the MgO particles showed a comparable extent of reaction between the 0 and 2.5 wt.% NC samples. Although brucite was detected by TGA, it could not be clearly identified, unlike portlandite, which typically forms large, identifiable structures or exhibits preferential formation in PC-based systems. This may be due to the brucite being interspersed within M-S-H, making it too fine to resolve. In carbonate-containing samples, the brucite was also poorly crystalline, further complicating its identification.

**Fig. 6:**
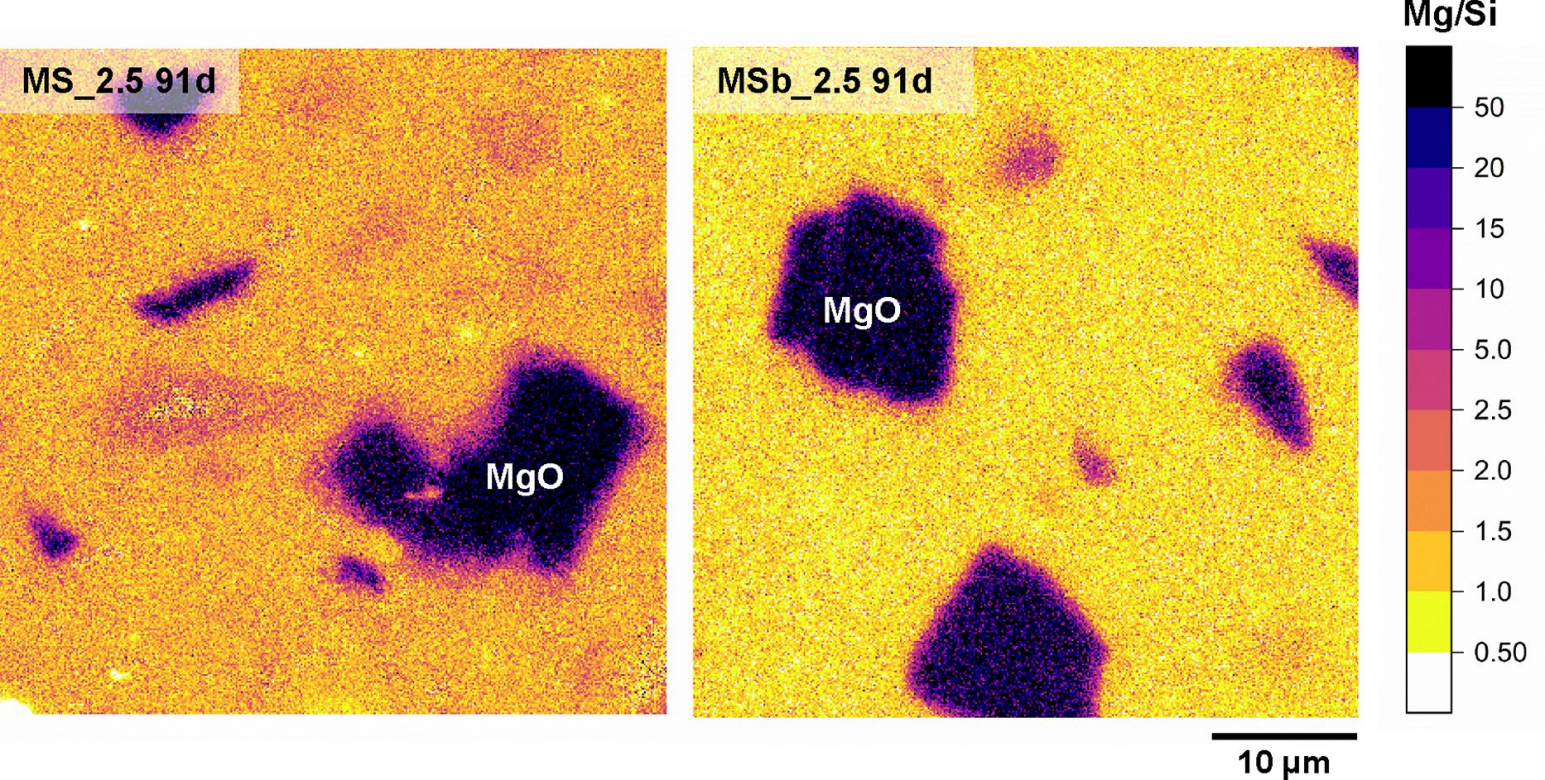
EDS mapping of Mg/Si ratio in mixed and ball-milled samples with 2.5 wt.% NC cured for 91 days. Corresponding BSE images are shown in Fig. S10

Figure 6 presents the EDS mapping of the Mg/Si atomic ratio in selected regions of interest in MS_2.5 (mixed) and MSb_2.5 (ball-milled) samples at 91 days. A homogeneous distribution of Mg/Si in the matrix between MgO particles was observed in both samples. However, a clear difference was evident: the Mg/Si ratio in the matrix was higher in MS_2.5, with an average value of ~ 1.5, and lower in MSb_2.5, averaging ~ 1. The value for MS_2.5 was close to Mg/Si=1.45, as calculated from mass balance, based on XRD Rietveld analysis and ^29^Si MAS NMR results in [17]. Although the MgO particles exhibited a thicker outer rim (or shell) in MS_2.5 compared to MSb_2.5, suggesting a greater extent of reaction, the Mg/Si ratio within the rim ranged from 2.5 to 15 in both cases. The porous microstructure of the mixed samples may have facilitated higher mobility of Mg ions, and in turn a greater reaction extent [47].

### Compressive strength

**Fig. 7:**
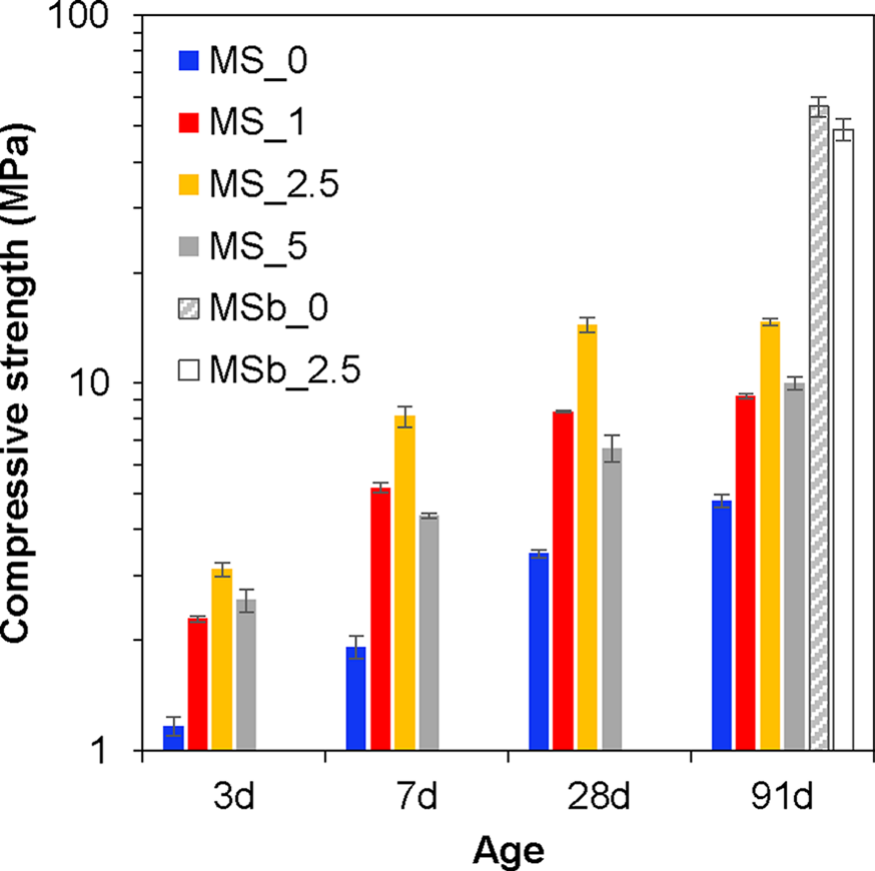
Compressive strength of all samples as a function of age. Error bars represent standard error. Data for mixed samples (MS_0 to MS_5) are reproduced from [17]

Figure 7 shows the compressive strength for all samples. The results for the mixed samples were previously reported in [17]. A clear influence of NC and age was observed in the mixed samples. At 3 days, the addition of 2.5 wt.% NC increased compressive strength by 166% relative to the sample with 0% NC. The enhancement in strength for the 2.5 wt.% NC sample was sustained across all ages. The ball-milled samples exhibited significantly higher compressive strengths than the mixed samples at 91 days (~ 10 × higher for 0 wt.% NC and ~ 3 × higher for 2.5 wt.% NC), despite having comparable solid phase assemblages (Fig. 2). No clear effect of NC was observed in the ball-milled samples. These findings suggest that the differences in compressive strength between the mixed and ball-milled samples are due to variations in in the physical microstructure (particle packing and porosity), rather than differences in the phase composition.

**Fig. 8:**
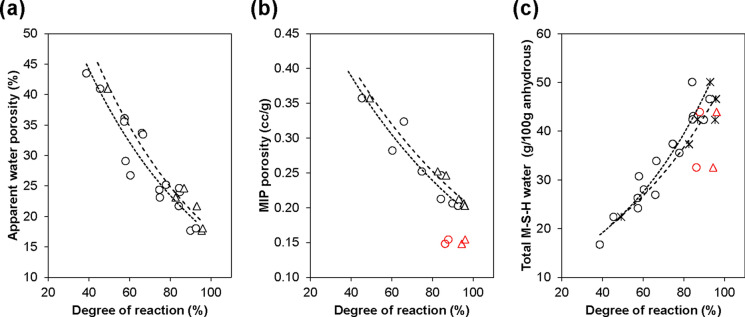
Correlation between the degrees of reaction of MgO (including brucite) (circles) and SF (triangles), and accessible porosities measured by **a** water absorption and **b** MIP, and **c** total water in M-S-H measured by TGA. Black markers represent mixed samples, while red markers represent ball-milled samples. Degrees of reaction for mixed samples are reproduced from [17]

Figure 8 shows the correlation between the degrees of reaction (DoR) of MgO (including crystalline brucite, which is deemed to have a limited contribution to strength [35]) and SF, as calculated from XRD and ^29^Si NMR data, respectively [17], with accessible porosities (Sect. 3.2.1), and total water in M-S-H (Sect. 3.1, Table 3). For the mixed samples, excellent correlations were observed, especially with the DoR of MgO. As expected, increasing DoR of MgO and SF led to reduced porosities (measured by both water absorption and MIP) and increased total water in M-S-H. However, the ball-milled samples did not seem to lie along on the same trend lines as the mixed samples. At a similar DoR, the ball-milled samples exhibited lower porosity, due to improved particle packing and a denser microstructure, although no clear trend was observed for the total water in M-S-H.

**Fig. 9:**
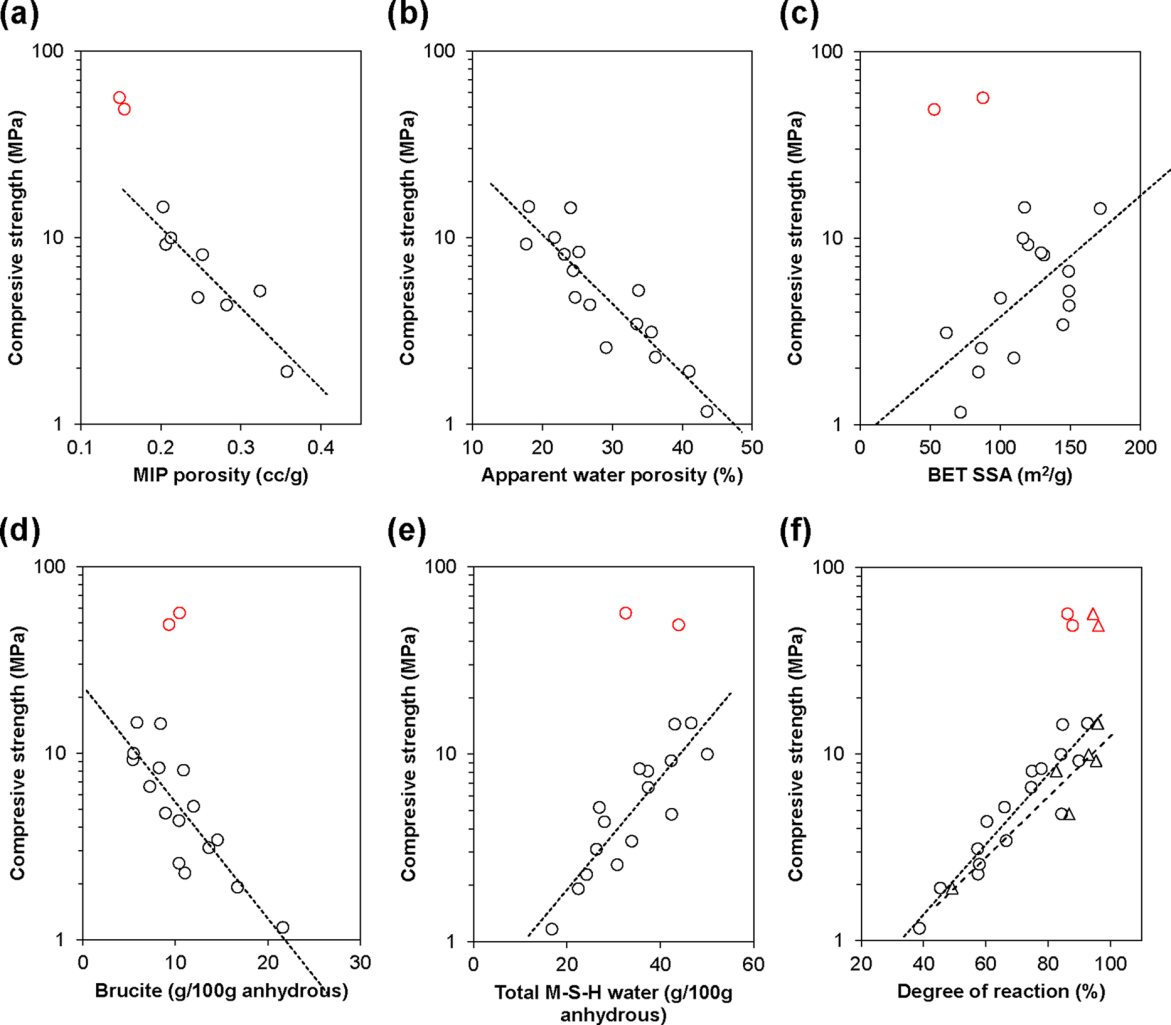
Correlation between compressive strength and (**a**, **b**) accessible porosities measured by MIP and water absorption, **c** BET pore specific surface area measured by N_2_ adsorption, **d**, **e** brucite and total water in M-S-H measured by TGA, and **f** degrees of reaction of MgO (including brucite) (circles) and SF (triangles). Black markers represent mixed samples, while red markers represent ball-milled samples. Degrees of reaction for mixed samples are reproduced from [17]

Figure 9 shows strong correlations between compressive strength and various parameters for the mixed samples, including porosities (measured by both MIP and water absorption), BET SSA, brucite content (crystalline and poorly crystalline HCB), total M-S-H water, and DoR of MgO and SF. Compressive strength decreased with increasing porosity, but increased with BET SSA, possibly due to the formation of a finer pore structure within M-S-H, analogous to C-S-H gel pores in PC systems, where SSA increases with hydration [29, 48]. Compressive strength also increased with decreasing brucite and increasing M-S-H contents, along with higher DoR of MgO and SF, confirming the greater binding contribution of M-S-H compared to brucite. This is despite German et al. [35] suggesting that poorly crystalline brucite in the form of HCB contributes more to strength than the crystalline form. The ball-milled samples did not appear to lie along the same trend lines as the mixed samples, except in the case of MIP porosity. At similar degrees of hydration and levels of hydrate formation, the ball-milled samples exhibited significantly higher compressive strength. This confirms the physical influence of particle packing and in turn the pore structure on compressive strength.

Future work could involve experimental determination of the packing density through normal consistency tests [49, 50], supported by characterisation of the binder’s overall particle size distribution, specific surface area, and density, to more rigorously quantify the effect of particle packing. Mathematical and computational modelling [50, 51] may also be employed to support the experimental observations. In this study, low-speed ball milling is presented as a laboratory-scale method to obtain well-dispersed paste samples, thereby enabling controlled investigation of the influence of MgO and silica fume dispersion on particle packing and microstructural development. In practical applications, the incorporation of aggregates and the use of superplasticisers are expected to aid dispersion, although this requires further investigation.

## Conclusions

Two distinct mixing techniques were employed to prepare M-S-H pastes using MgO and SF from different sources but with comparable properties, and varying concentrations of NC: (1) conventional Hobart-style paddle mixing, and (2) low-speed ball-mill mixing aimed at improving particle dispersion. The effects of these mixing techniques and additions of NC on the phase assemblage, microstructure and mechanical performance were systematically investigated. The key findings are:

- Conventional mixing resulted in poor dispersion of SF, producing a highly porous structure with large pore sizes, as observed by MIP. In contrast, ball-mill mixing significantly improved SF dispersion, leading to a dense microstructure with fine pores, although microcracks were observed under BSE microscopy.
- NC had a positive effect on the microstructure of conventionally mixed samples, with an optimum concentration 2.5 wt.%, resulting in the lowest porosity and smallest pore sizes, as observed by MIP. This effect was not evident in ball-milled samples, likely due to their already very dense microstructure induced by the ball-milling action, which limits the mobility of Mg ions and constrains further reaction, even in the presence of NC.
- Ball-milled samples disintegrated upon contact with water. This is an important observation that warrants further investigation. A plausible explanation for this behaviour is that the dense microstructure provides insufficient space for stress release upon drying and wetting. This behaviour was not observed in conventionally mixed samples, which were inherently more porous.
- Preliminary N_2_ adsorption analysis indicated an increase in pore specific surface area with NC and age for conventionally mixed samples, with most pores falling within 2–50 nm range. Further work is required to optimise the degassing protocol, and replicate measurements are necessary to confirm the findings.
- SEM–EDS analysis revealed a higher Mg/Si ratio in the hydrate matrix of conventionally mixed samples compared to ball-milled ones. This difference can be attributed to increased mobility of Mg^2^⁺ ions facilitated by the more porous microstructure of the conventionally mixed samples, as supported by the observation of larger hydration rims around MgO particles under BSE microscopy.
- Brucite content quantified by TGA agreed well with XRD Rietveld analysis for carbonate-free samples, but was higher for carbonate-containing samples, suggesting the presence of poorly crystalline brucite that may contain water and carbonate, and that cannot be detected by XRD but can be observed by TGA.
- Despite similar phase assemblages, ball-milled samples exhibited up to 10 × higher compressive strength than conventionally mixed samples. This improvement is attributed to their denser microstructure resulting from improved SF dispersion, highlighting the important physical role of particle packing in strength development. The effect of NC was less pronounced, consistent with the measured porosity.
- Strong correlations were identified between compressive strength, microstructure, and phase assemblage for the conventionally mixed samples. Strength increased with decreased porosity, greater M-S-H formation, and reduced brucite content. These findings confirm that M-S-H contributes more to mechanical strength than brucite.

## Supplementary Information

Below is the link to the electronic supplementary material.

Supplementary file1 (DOCX 10813 KB)

## Data Availability

Data will be made available on request.

## References

[1] Bonen D, Cohen MD (1992) Magnesium sulfate attack on portland cement paste–II. chemical and mineralogical analyses. Cem Concr Res 22(4):707–718

[2] De Weerdt K, Justnes H (2015) The effect of sea water on the phase assemblage of hydrated cement paste. Cem Concr Compos 55:215–222

[3] Zhang T, Vandeperre LJ, Cheeseman CR (2012) Magnesium-silicate-hydrate cements for encapsulating problematic aluminium containing wastes. J Sustain Cem-Based Mater 1(1–2):34–45

[4] Walling SA et al (2015) Structure and properties of binder gels formed in the system Mg(OH)_2_–SiO_2_–H_2_O for immobilisation of magnox sludge. Dalton Trans 44(17):8126–8137 25833071 10.1039/c5dt00877h

[5] Wang L et al (2019) Novel synergy of Si-rich minerals and reactive MgO for stabilisation/solidification of contaminated sediment. J Hazard Mater 365:695–706 30472455 10.1016/j.jhazmat.2018.11.067

[6] Bernard E et al (2023) Mgo-based cements–current status and opportunities. RILEM Tech Lett 8:65–78

[7] Scott A et al (2021) Transformation of abundant magnesium silicate minerals for enhanced CO_2_ sequestration. Commun Earth Environ 2(1):25

[8] Chu SH, Yang EH, Unluer C (2023) Chemical synthesis of magnesium oxide (MgO) from brine towards minimal energy consumption. Desalination 556:116594

[9] Badjatya P et al (2022) Carbon-negative cement manufacturing from seawater-derived magnesium feedstocks. Proc Natl Acad Sci U S A 119(34):e2114680119 35972958 10.1073/pnas.2114680119 PMC9407650

[10] Nied D et al (2016) Properties of magnesium silicate hydrates (M-S-H). Cem Concr Res 79:323–332

[11] Bernard E et al (2019) Characterization of magnesium silicate hydrate (M-S-H). Cem Concr Res 116:309–330

[12] Simoni M et al (2023) Reaction mechanisms, kinetics, and nanostructural evolution of magnesium silicate hydrate (M-S-H) gels. Cem Concr Res 174:107295

[13] Wei J, Chen Y, Li Y (2006) The reaction mechanism between MgO and microsilica at room temperature. J Wuhan Univ Technol-Mater Sci Ed 21(2):88–91

[14] Zhang T, Vandeperre LJ, Cheeseman CR (2014) Formation of magnesium silicate hydrate (M-S-H) cement pastes using sodium hexametaphosphate. Cem Concr Res 65:8–14

[15] Tonelli M et al (2019) Effect of phosphate additives on the hydration process of magnesium silicate cements. J Therm Anal Calorim 138(5):3311–3321

[16] Bernard E et al (2022) Effect of carbonates on the formation of magnesium silicate hydrates. Mater Struct 55(7):183

[17] Bernard E et al (2024) Insights on the effects of carbonates and phosphates on the hydration of magnesia (alumino-)silicate cements. Appl Geochem 167:106001

[18] Shah V, Scott A (2021) Hydration and microstructural characteristics of MgO in the presence of metakaolin and silica fume. Cem Concr Compos 121:104068

[19] Szczerba J et al (2013) Influence of time and temperature on ageing and phases synthesis in the MgO–SiO_2_–H_2_O system. Thermochim Acta 567:57–64

[20] Roosz C et al (2015) Crystal structure of magnesium silicate hydrates (M-S-H): the relation with 2:1 Mg–Si phyllosilicates. Cem Concr Res 73:228–237

[21] Bernard E, Nguyen H (2024) Magnesium silicate hydrate (M-S-H) stability under carbonation. Cem Concr Res 178:107459

[22] Sreenivasan H et al (2024) A critical review of magnesium silicate hydrate (M-S-H) phases for binder applications. Cem Concr Res 178:107462

[23] Tran HM, Scott A (2017) Strength and workability of magnesium silicate hydrate binder systems. Constr Build Mater 131:526–535

[24] Shah V, Dhakal M, Scott A (2022) Long-term performance of MgO–SiO2 binder. Mater Struct 55(2):60

[25] Dewitte C et al (2022) Chemical and microstructural properties of designed cohesive M-S-H pastes. Materials 15(2):547 35057269 10.3390/ma15020547 PMC8782006

[26] Bernard E et al (2020) Micro-X-ray diffraction and chemical mapping of aged interfaces between cement pastes and Opalinus Clay. Appl Geochem 115:104538

[27] Winnefeld F, Schöler A, Lothenbach B (2016) Sample preparation. Pract Guide Microstruct Anal Cem Mater 1:1–36

[28] Lothenbach B, Durdzinski P, De Weerdt K (2016) Thermogravimetric analysis. Pract Guide Microstruct Anal Cem Mater 1:177–211

[29] Zhang Z, Scherer GW (2019) Evaluation of drying methods by nitrogen adsorption. Cem Concr Res 120:13–26

[30] Brunauer S, Emmett PH, Teller E (1938) Adsorption of gases in multimolecular layers. J Am Chem Soc 60(2):309–319

[31] Rouquerol J, Llewellyn P, Rouquerol F (2007) Is the bet equation applicable to microporous adsorbents? In: Llewellyn PL et al (eds) Studies in surface science and catalysis. Elsevier, pp 49–56

[32] Desmurs L et al (2022) Determination of microporous and mesoporous surface areas and volumes of mesoporous zeolites by corrected t-plot analysis. ChemNanoMat 8(4):e202200051

[33] Barrett EP, Joyner LG, Halenda PP (1951) The determination of pore volume and area distributions in porous substances. I. computations from nitrogen isotherms. J Am Chem Soc 73(1):373–380

[34] Palacios M et al (2016) Laser diffraction and gas adsorption techniques. A practical guide to microstructural analysis of cementitious materials. CRC Press, pp 445–480

[35] German A et al (2023) Hydrous carbonate-containing brucite (HCB) in MgO/hydromagnesite blends. Cem Concr Res 173:107304

[36] Jansen D et al (2024) Stacking faults of the hydrous carbonate-containing brucite (HCB) phase in hydrated magnesium carbonate cements. Cem Concr Res 175:107371

[37] Frost RL, Kloprogge JT (1999) Infrared emission spectroscopic study of brucite. Spectrochim Acta A Mol Biomol Spectrosc 55(11):2195–2205

[38] Wu Z et al (2019) Anomalous water absorption in cement-based materials caused by drying shrinkage induced microcracks. Cem Concr Res 115:90–104

[39] Zhang T et al (2016) Control of drying shrinkage in magnesium silicate hydrate (m-s-h) gel mortars. Cem Concr Res 88:36–42

[40] Zhang T, Fu H, Han J (2023) Deformation mechanisms of magnesium silicate hydrate cement with a shrinkage-reducing admixture under different curing conditions. Minerals 13(4):563

[41] Nurcahya N et al., (2025) Microstructure and transport properties of magnesia (alumino-) silicate cement mortars, In: RILEM Spring Convention 2025, RILEM: Mendrisio

[42] Kurihara R, Maruyama I (2022) Surface area development of Portland cement paste during hydration: direct comparison with 1H NMR relaxometry and water vapor/nitrogen sorption. Cem Concr Res 157:106805

[43] Liu S et al (2021) Adsorption of lead ion from wastewater using non-crystal hydrated calcium silicate gel. Materials 14(4):842 33578734 10.3390/ma14040842 PMC7916452

[44] Mantellato S, Palacios M, Flatt RJ (2016) Impact of sample preparation on the specific surface area of synthetic ettringite. Cem Concr Res 86:20–28

[45] Martini F et al (2018) Monitoring the hydration of MgO-based cement and its mixtures with Portland cement by ^1^H NMR relaxometry. Microporous Mesoporous Mater 269:26–30

[46] Scrivener KL (2004) Backscattered electron imaging of cementitious microstructures: understanding and quantification. Cem Concr Compos 26(8):935–945

[47] Jug K, Heidberg B, Bredow T (2007) Cyclic cluster study on the formation of brucite from periclase and water. J Phys Chem C Nanomater Interfaces 111(35):13103–13108

[48] Garci Juenger MC, Jennings HM (2001) The use of nitrogen adsorption to assess the microstructure of cement paste. Cem Concr Res 31(6):883–892

[49] Lecomte A, Mechling J-M, Diliberto C (2009) Compaction index of cement paste of normal consistency. Constr Build Mater 23(10):3279–3286

[50] Knop Y, Peled A, Cohen R (2014) Influences of limestone particle size distributions and contents on blended cement properties. Constr Build Mater 71:26–34

[51] Wang M, Al-Tabbaa A, Wang W (2019) Improving discrete particle packing models for the microstructural formation simulation of Portland cement. Constr Build Mater 229:116841

