# Supplementary material for: Microstructure of magnesium silicate hydrate pastes influenced by carbonate and mixing method
Source: Mater Struct. 2025 Oct 23;58(9):300. doi: 10.1617/s11527-025-02837-0 (PMC12549767; doi:10.1617/s11527-025-02837-0)
Supplement: Supplementary file 1 — Supplementary file1 (DOCX 10813 KB) [file 11527_2025_2837_MOESM1_ESM.docx]

**Microstructure of magnesium silicate hydrate pastes influenced by carbonate and mixing method**

M.H.N. Yio^1*^, E. Bernard^1&2^, C. Dewitte^2^, H. Chen^1^, T.C. Chan^1^, R.J. Myers^1^

*^1^Department of Civil and Environmental Engineering, Imperial College London, SW7 2AZ, United Kingdom*

*^2^ Empa, Swiss Federal Laboratories for Materials Science and Technology, Laboratory for Concrete and Asphalt, 8600 Dübendorf, Switzerland*

**Corresponding author: marcus.yio@ic.ac.uk*

**Supplementary information**

|  |
| --- |
| **Fig. S1: Particle size distribution of (a) MgO and (b) silica fume measured by laser diffraction.** |

| **** |
| --- |
| **Fig. S2: (CBW_MSH_+PAW_M-S-H_) /CBW_M-S-H_ derived from TGA data, plotted against NC content to identify outliers.** |

| **** |
| --- |
| **Fig. S3: TGA curves of 3-day and 91-day samples. Note that the mass loss at 520°C observed for MS_0 and MS_1 at 91 days could not be attributed to any known phase. It is unlikely to be related to hydromagnesite, as MS_0 contained no NC. MS_x: conventional mixing; MSb_x: ball-mill mixing (x = NC concentration).** |

**Table S1: Quantified CBW_brucite_, CBW_M-S-H_ and PAW_M-S-H_ from TGA data. All results were normalised to 100 g anhydrous materials. MS_x: conventional mixing; MSb_x: ball-mill mixing (x = NC concentration).**

| **Sample ID** | **CBW_brucite_** | | | | **CBW_M-S-H_** | | | | **PAW_M-S-H_** | | | |
| --- | --- | --- | --- | --- | --- | --- | --- | --- | --- | --- | --- | --- |
|  | **3d** | **7d** | **28d** | **91d** | **3d** | **7d** | **28d** | **91d** | **3d** | **7d** | **28d** | **91d** |
| MS_0 | 6.68 | 5.17 | 4.50 | 2.76 | 7.55 | 10.3 | 13.2 | 16.9 | 9.18 | 12.2 | 20.7 | 25.5 |
| MS_1 | 3.41 | 3.70 | 2.54 | 1.67 | 8.21 | 9.52 | 14.2 | 16.6 | 16.0 | 17.4 | 21.4 | 25.7 |
| MS_2.5 | 4.45 | 3.36 | 2.59 | 1.81 | 7.66 | 10.8 | 14.1 | 15.2 | 18.6^a^ | 26.6 | 29.0 | 31.4 |
| MS_5 | 3.22 | 3.22 | 2.23 | 1.87 | 9.12 | 8.38 | 11.5 | 13.9 | 21.6 | 19.7 | 25.9 | 36.1^a^ |
| MSb_0 | - | - | - | 2.38 | - | - | - | 14.4 | - | - | - | 18.2 |
| MSb_2.5 | - | - | - | 1.96 | - | - | - | 13.4 | - | - | - | 30.5 |

^a^ Estimated from Fig. S4 based on total water in M-S-H = 3.25 × CBW_M-S-H_

| 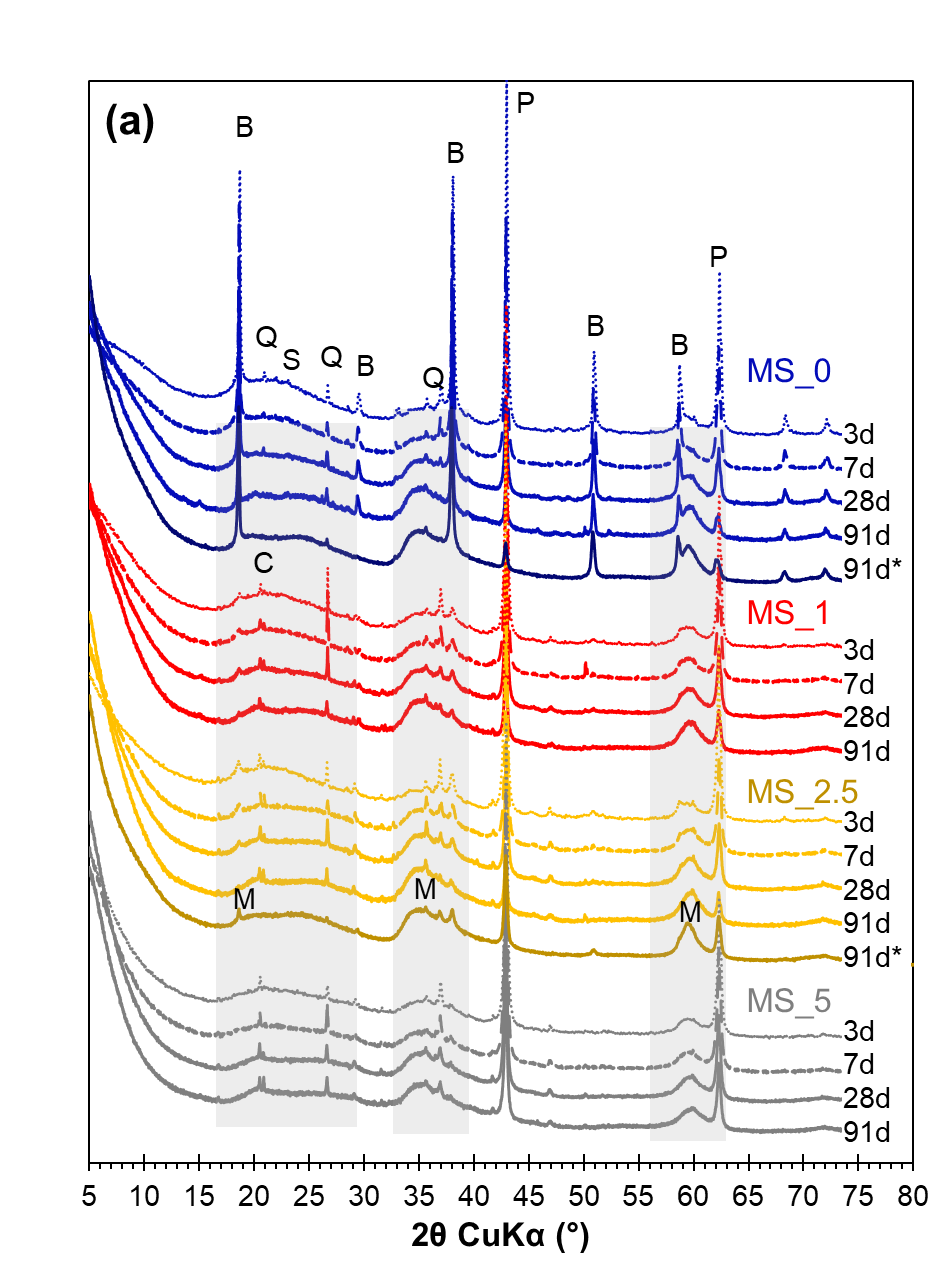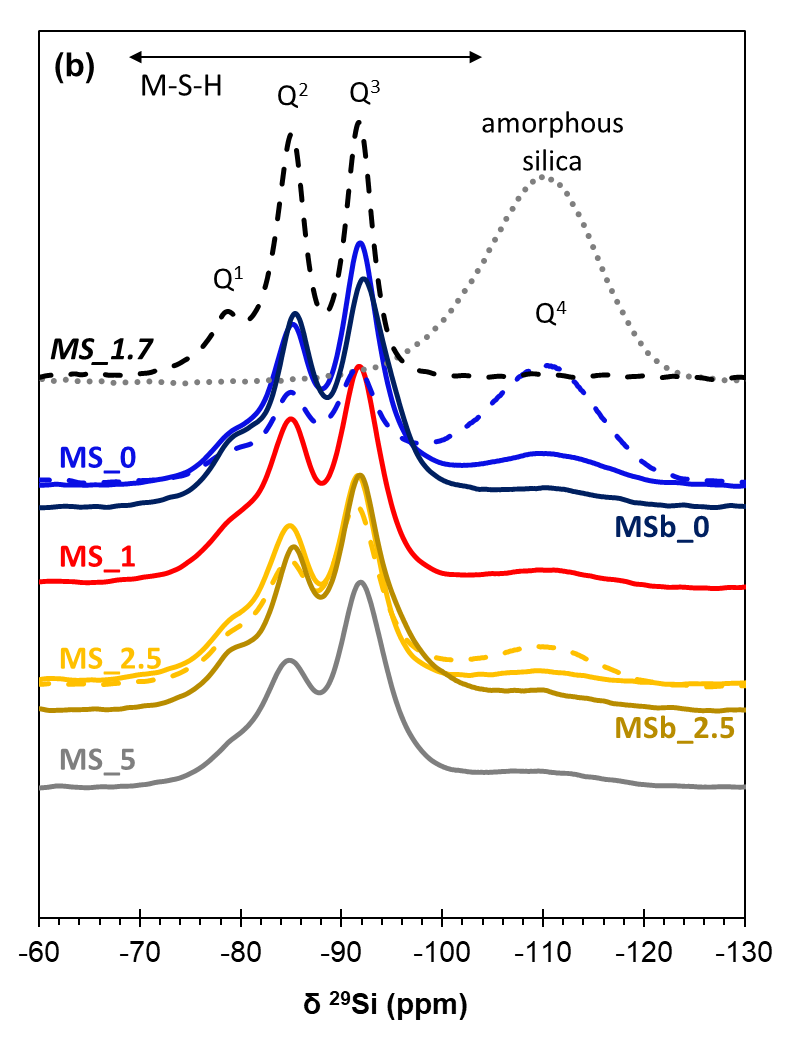 |
| --- |
| **Fig. S4: (a) XRD patterns, * = MSb samples and (b) ^29^Si MAS NMR spectra for all samples. Data for the mixed samples (MS_0 to MS_5) are reproduced from [17]. In (b), dashed lines represent 7-day samples, while solid lines correspond to 91-day samples. MS_1.7 is a reference M-S-H with 1.7 wt.% NC aged for 6 months taken from [16].** |

**Table S2: Relative amounts (wt.%) of Q^n^ silicon species obtained by deconvolution of the ^29^Si MAS NMR spectra shown in Figure S5b. Data for the mixed samples (MS_0 to MS_5) are reproduced from [17].**

| **Age** | **Sample ID** | **M-S-H** | | | **Amorphous silica (unreacted MS)** |
| --- | --- | --- | --- | --- | --- |
|  |  | **Q^1^** | **Q^2^** | **Q^3^** | **Q^4^** |
| 7 days | MS_0 | 10 | 13 | 26 | 51 |
|  | MS_2.5 | 13 | 24 | 46 | 17 |
| 91 days | MS_0 | 11 | 27 | 49 | 13 |
|  | MS_1 | 18 | 29 | 48 | 5 |
|  | MS_2.5 | 20 | 29 | 47 | 4 |
|  | MS_5 | 10 | 25 | 58 | 7 |
|  | MSb_0 | 13 | 26 | 56 | 5 |
|  | MSb_2.5 | 10 | 19 | 67 | 4 |

| **** |
| --- |
| **Fig. S5: Comparison of brucite content quantified by XRD and TGA. Error bars for MS_0 and MSb_0 represent a 10% error, while those for the remaining samples represent a 2% error. MS_x: conventional mixing; MSb_x: ball-mill mixing (x = NC concentration).** |

| **** |
| --- |
| **Fig. S6: Cumulative water absorption plot for mixed samples cured for 91 days. MS_x: conventional mixing (x = NC concentration).** |

| **** |
| --- |
| **Fig. S7: Comparison of measured porosities and thermodynamic model predictions of free water content in the systems (reproduced from [17]). Dashed lines serve as a visual guide.** |

| **** |
| --- |
| **Fig. S8: Instantaneous disintegration of a dried paste sample upon contact with water. The sample was prepared with a different MgO and well-dispersed SF (Mg/Si 1.5, w/b 0.75, 1.5 wt.% NC), cured at 21^o^C, > 99% RH for 91 days.** |

| **** |
| --- |
| **Fig. S9: Comparison of BET SSA values measured in this study with those reported in the literature. MS: conventional mixing; MSb: ball-mill mixing.** |

|  |
| --- |
| **Fig. S10: Selected regions of interest in MS_2.5 and MSb_2.5 for EDS mapping at 91 days.** |
